# Serological Evaluation of Anti-*Toxoplasma gondii* Antibodies in Patients with Acute Leukemia and Lymphoma through Chemotherapy

**Published:** 2020

**Authors:** Fatemeh TABATABAIE, Taher ELMI, Majid KHANMOHAMMADI, Lame AKHLAGHI, Mahmoud MAHAMI-OSKOUEI, Mehdi ARSHADI

**Affiliations:** 1. Department of Parasitology and Mycology, School of Medicine, Iran University of Medical Sciences, Tehran, Iran; 2. Department of Laboratory Sciences, Marand Branch, Islamic Azad University, Marand, Iran; 3. Department of Parasitology and Mycology, Faculty of Medicine, Tabriz University of Medical Sciences, Tabriz, Iran; 4. Al-Zahra Hospital, Tabriz University of Medical Sciences, Tabriz, Iran

**Keywords:** *Toxoplasma gondii*, Leukemia, Lymphoma, Immunoglobulin G, Immunoglobulin M

## Abstract

**Background:**

*Toxoplasma gondii* is a protozoan parasite that belongs to the family Coccidae. We aimed to evaluate IgG avidity and the changes of anti-*Toxoplasma* immunoglobulins M (IgM) and G (IgG) in patients with acute leukemia and lymphoma.

**Methods:**

Ninety eight patients with Acute myeloid leukemia (AML), Acute Lymphoblastic Leukemia (ALL) and lymphoma, selected from patients referring to Imam Reza Hospital of Tabriz (38°04′N 46°18′E), in terms of the presence of anti-*Toxoplasma* IgM, IgG, IgG avidity antibodies and the major risk factors were evaluated.

**Results:**

The results of pre-chemotherapy evaluation showed that of the examined patients, only two cases, one patient with ALL and another patient with lymphoma, had a positive IgM titer. Overall, 46 cases had positive IgG titers, including 20 patients with AML, 15 patients with ALL and 11 patients with lymphoma. Three (3.06%) patients were positive for anti-*T. gondii* IgM and one of them was with new infection of toxoplasmosis in lymphoma patients. The post-chemotherapy IgG titer evaluation showed 46 [46.9% (95% CI 37.4–56.7)] positive IgG cases that this result was similar to the result of pre-treatment phase. One [1% (95% CI 0.2–5.6)] positive IgG avidity case was detected using ELISA method, in a patient with lymphoma whose IgM was also positive. There was no significant difference between the type of leukemia and the history of contact with cat.

**Conclusion:**

Performing specialized tests to diagnose toxoplasma infection before starting treatment, in immunodeficiency patients who undergo chemotherapy, is necessary; therefore, these tests should be considered in therapeutic protocols.

## Introduction

*Toxoplasma gondii* is an intracellular opportunistic coccidian parasite transmitted from animal to human via oocysts and tissue cysts. Toxoplasmosis refers to the disease caused by this parasite reported worldwide, especially in industrialized and developed countries (1–3). Nearly 60% of the adult population is seropositive and all types of mammals can become infected with this parasite. There is no specific clinical symptom in chronic toxoplasmosis in individuals with a healthy and effective immune system and only presence of IgG antibody is indicative of their previous infection.

Lymphadenopathy, Mononucleosis -like syndrome and Chorioretinitis are reported in 10%–20%, 1% and small number of cases, respectively. Lymphadenopathy, mononucleosis -like syndrome and chorioretinitis have been reported in 10%–20%, 1% and a small number of these patients, respectively. However, leukemia, lymphomas and the use of immunosuppressive drugs are the major concerns affected on opportunistic parasitic infections in pregnant mothers, immunocompromised individuals, patients with diarrhea and also in the postpartum period. Leukemia and cystic lymphoma of the brain are reactivated and cause encephalitis and other brain lesions in immunocompromised patients, which ultimately lead to the death of the patient (4–8). Leukemias and lymphomas include cancers that originate from blood-forming cells and immune cells. Lymphoma is the preliminary step in the development of blood cell cancers, which affects lymphocytes. It leads to abnormal growth and function of lymphocytes.

Hodgkin’s and non-Hodgkin’s lymphomas are two major types of lymphoma (9–11). The individuals with this disease are susceptible to opportunistic infections, including parasitic infections. Today, these infections account for the major mortality rate in these patients (11–14). *T. gondii* infection or toxoplasmosis is one of the most important protozoan infections in immunocompromised, lymphoma and leukemia patients which cause mortality among patients. Therefore, it is important in this regard. *T. gondii* encephalitis is an important opportunistic infection, and its symptoms appear in the central and white-gray areas of the brain (15–17). Specific antibodies in serum of patients with toxoplasmosis are detectable shortly after the infection; the main diagnosis of this disease is currently based on serologic methods (18). The ELISA method can be used to detect antigens and antibodies that show high affinity and strong binding power to each other. This method is used to measure both types of IgG and IgM antibodies. Hedman et al. devised a new method based on the tendency of immunoglobulin binding to polyvalent antigens of *T. gondii* and the high concentrations of urea were used to differentiate the high tendency of immunoglobulin. Today, this binding power is called avidity (19). The IgG avidity, in the new infection of toxoplasmosis (early stage), is much lower than the old (previous) IgG avidity. According to the conducted studies, today, avidity below 40% and higher than 50% indicate the primary (active) and previous infections, respectively (20, 21).

Considering the major role of this opportunistic parasitic disease in the mortality of immunocompromised, lymphoma and leukemia patients and also patient undergoing chemotherapy, the present study aimed at the serological evaluation of anti-toxoplasmosis antibodies in acute leukemia and lymphoma before and after chemotherapy in patients referring to medical centers in Tabriz.

## Materials and Methods

### Ethical Consideration

This study has been approved by research council of Iran University of Medical Sciences (IR.IUMS.FMD.REC1396.32565). The selected patients signed informed consent forms after being informed about the nature of the study, including the objectives and laboratory procedures.

### Study type, population and risk factors

We performed a cross-sectional study in Imam Reza Hospital of Tabriz, East Azerbaijan Province, northwest of Iran, from Jun 2015 to Jun 2016. The present one year-clinical trial was performed on 98 patients with acute myeloid leukemia (AML), acute lymphoblastic leukemia (ALL), and lymphoma (Table 1).

**Table 1:** Frequency distribution of Sex, Age, Undercooked meat-eating and Contact with Cat variables in patients

***Variable***	***Frequency No (%)***	***Toxoplasma***		**P- *value***
		***Positive (%)***	***Negative (%)***	
Gender				
Male	34 (34.7)	16 (47)	18 (53)	*P*= 0.492
Female	64 (65.3)	30 (46.8)	34 (53)	
Total	98 (100.0)	46 (46.9)	52 (53)	
Age (yr)				
≤20	16 (14.2)	6 (37.5)	10 (62.5)	
21–35	25 (22.4)	12 (48)	13 (52)	*P*= 0.098
36–50	38 (37.7)	18 (47.3)	20 (52.7)	
>50	19 (16.3)	10 (52.6)	9 (47.4)	
Total	98 (100.0)	46 (46.9)	52 (53)	
Undercooked meat				
Yes	31 (31.6)	20 (58.8)	14 (41.2)	P= 0.061
No	67 (68.4)	26 (40.6)	38 (59.4)	
Total	98 (100.0)	46 (46.9)	52 (53)	
Contact with Cat				
Yes	37 (37.8)	19 (51.3)	18 (48.7)	*P*= 0.058
No	61 (62.2)	27 (44.3)	34 (55.7)	
Total	98 (100.0)	46 (46.9)	52 (53)	

Patients who were willing to cooperate and submitted their informed consent form enrolled in this study.

A questionnaire was presented to patients, in which information such as age, gender, history of contact with a cat and consumption of uncooked meat was written. Individuals who did not fully cooperate until the end of the study and those who were only at the beginning of chemotherapy or in the final stages of treatment were excluded from the study. The diagnosis of leukemia and lymphoma cases was carried out by a hematologist-oncologist in specialized clinics of Tabriz University of Medical Sciences. The patients underwent blood drawn before and two months after chemotherapy and 5 ml blood samples were collected from each patients and then collecting blood centrifuged and prepared serum samples stored in freeze condition.

### Serological assay for diagnosis of anti-Toxoplasma antibodies

To evaluate the anti-*Toxoplasma* antibodies, we analyzed all the serum samples using chemiluminescence immunoassay (CLIA) with Diasorin Liaison calibrated analyzer (Germany) by a commercially available kit (Diasorin, Italy), Both tests (IgG and IgM) were performed, following the instructions of the manufacturer.

The values of more than 8.8 and 8 international units IU/mL were considered positive for IgG and IgM, respectively. The ELISA was used to evaluate the IgG and IgM antibody titers and also the avidity of IgG antibodies against *Toxoplasma* using commercial kit (ACON Laboratories, Inc. USA) on the market.

The values of more than 11 international units IU/mL were considered positive for IgG and IgM, respectively. Blood samples were collected from patients two times before and after chemotherapy (about two months). After centrifugation, sera were kept at −20 °C until used. In this study, ELISA kit made by Genesis Company of England was used. According to the manufacturer’s instructions, IgG titers <15 and ≥ 15 IU/ml were considered negative and positive, respectively and also IgM titers < 1 and ≥ 1 IU/ml were considered as negative and positive cases, respectively. To ensure the results, all serum samples were examined for IgG and IgM antibody titers by CLIA (a new chemiluminescent assay) using IgG and IgM *T. gondii* kit (Diasorin, LIAISON USA).

According to the manufacturer’s instruction, IgG antibody titers < 7.2, 7.2–8.8% and ≥ 8.8% IU/ml were considered as negative, borderline and positive cases, respectively. Moreover, IgM antibody titers < 6%, 6%–8% and ≥ 8% IU/ml were considered as negative, borderline and positive cases, respectively.

### Titration of anti-Toxoplasma IgG avidity using ELISA

In this study, IgG avidity ELISA test was used to determine the acute and chronic infection of disease in patients with leukemia and lymphoma. The avidity of IgG antibodies was evaluated and the percentage of avidity for each positive IgG calculated using ELISA Kit (Biomerieux, France) and VIDAS aparatus according to the manufacturer’s instructions. An IgG avidity >40% indicated past infection and also IgG avidity <40% indicated recent toxoplasmosis infection.

### Statistical analysis of the results

SPSS software ver. 24 (Inc., Chicago, IL) was used for the statistical analysis. A descriptive analysis was performed to evaluate the frequency of the variables and *T. gondii* antibodies. Possible associations were identified using Chi-squared tests at a significance level of 0.05.

## Results

Of the 98 patients who were recruited in this study, 34 (34.7%) were female and 64 (65.3%) were male, with the age ranges of 14 to 65 yr and a mean of 35.4 yr (SD= 10) (Table 1). Moreover, 46 (46.9%), 30 (30.6%) and 22 (22.5%) of patients had AML, ALL and lymphoma, respectively (Table 2).

**Table 2:** The number of patients with AML, ALL and lymphoma for each sex

***Patients***	***Male, No (%)***	***Female, No (%)***	***Total***
AML	18 (18.4)	28 (28.6)	46 (47)
ALL	11(11.2)	19 (19.4)	30 (30.6)
Lymphoma	5 (5.1)	17 (17.3)	22 (22.4)
Total	34 (34.7)	64 (65.3)	98 (100.0)

### Risk Factors

According to the information on the questionnaire, 88 individuals had a history of contacting with cats. There was no significant difference between the type of leukemia and the history of contact with cat.

Among the patients with AML, 28 were female [60.9% (95% CI 46.5 – 73.6)] and 18 were male [39.1% (95% CI 26.4 – 53.3)], and with ALL, 19 were female [63.5% (95% CI 45.5–78.1)] and 11 were male [36.7% (95% CI 21.9 – 54.5)] and also there were 17 female [73.3% (95% CI 56.6 –89.9) ] and 5 male [22.7% (95% CI 10. – 43.4)] patients with lymphoma. There was also no significant difference between diseases, gender.

### Serologic evaluation before chemotherapy

The results of pre-chemotherapy evaluation showed that of the 98 patients examined, only two cases including one patient with ALL and another with lymphoma, had a positive IgM titre of above 1 IU/ml and 46 [46.9% (95% CI 37.4–56.7)] cases had positive IgG titers, 20 [43.5% (95% CI 30.2–57.8)], 15 [50% (95% CI 33.2–66.8)] and 11 [50% (95% CI 30.7–69.3)] of whom were patients with AML, ALL and lymphoma, respectively.

The results of pre-chemotherapy evaluation showed that only 2 (2.04%) of the 98 examined patients (one patient with ALL and another with lymphoma), had a positive IgM titer (>1 IU/ml) and 46 cases (46.9%) had positive IgG titers (>15 IU/ml), Out of these 46 patients, 20, 15 and 11 were AML, ALL and lymphoma, respectively.

### Serologic evaluation after chemotherapy

The results of the post-chemotherapy investigation revealed that 3 [3.06% (95% CI 1–8.6)] patients were positive for anti-Toxoplasma IgM and one of them was with new infection of toxoplasmosis in lymphoma patients. The post-chemotherapy IgG titer evaluation showed 46 [46.9% (95% CI 37.4–56.7)] positive IgG cases that this result was similar to the result of pre-treatment phase (Table 3).

**Table 3:** IgG and IgM positive cases (anti-Toxoplasma antibodies) and also IgG avidity in patients with leukemia and lymphoma before and after chemotherapy using ELISA

***Methods***	***Before chemotherapy No (%)***	***After chemotherapy No (%)***
ELISA / IgM	2 (2.04)	3 (3.06)
ELISA / IgG	46 (46.9)	46 (46.9)
ELISA / IgG avidity	0	1 (1.02)

The results of Chemiluminescence assay confirmed the results of ELISA. Therefore, 46 positive IgG cases were reported before treatment. The IgM positive cases, similar to the ELISA test, were also reported in 2 cases before treatment and in 3 cases after treatment (Table 4).

**Table 4:** IgG and IgM positive cases (anti-*Toxoplasma* antibodies) in patients with leukemia and lymphoma before and after chemotherapy with chemiluminescence

***Patients***	***No***	***IgG Positive***		***IgM Positive***	
		***Before treatment No (%)***	***After treatment No (%)***	***Before treatment No (%)***	***After treatment No (%)***
AML	46	20 (43.5)	20 (43.5)	0	0
ALL	30	15 (50)	15 (50)	1 (3.33)	1 (3.33)
Lymphoma	22	11 (50)	11 (50)	1 (4.5)	2 (9.1)
Total	98	46 (46.9)	46 (46.9)	2 (2.04)	3 (3.06)

According to the results obtained by the IgG avidity ELISA, all samples had avidity >40 before chemotherapy, but after the treatment, a positive case [1% (95% CI 0.2–5.6)] was identified in a patient with lymphoma whose IgM was also positive, with an IgG avidity below 40 that indicated reinfection with Toxoplasma (Table 2). All data were obtained from the duplicate samples, and the experimental procedure was repeated at least three times with identical results.

## Discussion

Our findings showed that in patients with ALL and lymphoma, the number of individuals who had anti- *Toxoplasma* IgG antibodies, before and after treatment, was 46.9%, and pre and post-treatment positive IgM cases were also observed.

Furthermore, a lymphoma patient was found with positive IgG avidity and IgM antibodies, which indicated a new infection in this patient.

Toxoplasmosis has long been recognized as an opportunistic infection in immunocompromised patients. It is the third leading cause of death in immunocompromised patients after pneumocystis and cryptosporidium, which has increased the importance of the issue. Among the causes of encephalitis in immunocompromised individuals, 20%–48% of cases are related to *T. gondii* and encephalitis is one of the major causes of mortality and disability in immunocompromised patients (11, 15, 18).

For instance, in one case reported in Ahvaz, Investigation of anti- *Toxoplasma* IgM antibodies using ELISA method in two patients treated with chemotherapy drugs, showed that the two patients had toxoplasmosis and both of them died one year later, due to their weak immune systems and activation of toxoplasmosis (22).

The diagnosis of *Toxoplasma* infection in patients with weak and ineffective immune systems is important and necessary. The most common clinical symptoms of secondary toxoplasmosis, in patients with immunodeficiency including lymphoma and leukemia, are fever, pneumonia, headache and dizziness.

Secondary toxoplasmosis in these patients is confused with many cases such as pneumonia, sepsis and encephalitis (8, 9, 11).

The prevalence of IgG antibodies to *Toxoplasma* (IgG-anti-*Toxoplasma*) was 42.8%, which is close to that in the present study (23). In Iran, 51.9% of thalassemia patients and 34.8% of healthy controls were positive for anti-*Toxoplasma* IgG antibodies and in terms of anti-*Toxoplasma* IgM antibody, 3.4% of patients with thalassemia and 2.1% of healthy individuals were positive (24). In various parts of the world, the most *T. gondii* infections in Brazil (80%) and the lowest in China (8%) have been reported. The difference in *Toxoplasma* infection rates in Iran, China and Brazil can be due to differences in weather, health condition, culture, customs and food habits (2). The increase in the course of the illness and the use of immunosuppressive drugs are expected to increase the risk of recurrence of toxoplasmosis (25). In the present study, there was no significant difference between duration of disease and recurrence, which conforms to another study on patients with immunodeficiency (26). In turkey IgG levels of anti-*T. gondii* antibodies were 63% in patients with cancer and 19.4% in healthy subjects, which were more than the IgG levels in present study, perhaps the reasons for this difference are age characteristics of the study group and the difference in the type of food consumed because of the difference in culture (27).

Anti-*Toxoplasma* antibodies, using ELISA was investigated, in patients with various types of neoplasia as a group of individuals who underwent immunosuppressive therapy with chemotherapy drugs.

The results of this study showed that more than 50% of these patients had anti-*Toxoplasma* IgG antibodies and finally, parasitological tests were performed to confirm toxoplasmosis in these patients. In the present study, the number of individuals with pre and post-treatment anti-*Toxoplasma* IgG antibody titers was 46.9%, which is in agreement with other studies (24, 27). In addition, in another study, an increase in antibodies in hemodialysis patients with chronic renal failure, by using ELFA technique was reported (28).

IgG and IgM antibodies were investigated in cancer patients. A large number of individuals had a positive IgG titer, which was valuable, but there was no significant difference between the positive IgM levels (29). The number of cases with IgG titers was lower than previous studies, attributed to the role of community culture, customs, traditions and food habits. A study carried out on renal transplant recipients in Iran. The participants were monitored from the third month to one year after transplantation for anti-*Toxoplasma* antibody titers. Toxoplasmosis is not a major risk factor in renal transplant patients (30). In the present study, the results also showed that toxoplasmosis is not considered as a major risk factor in individuals with acute leukemia and lymphoma, since the probability of infection recurrence is low in these patients due to the use of drug and chemotherapy. Several studies have referred to the positive and important role of avidity to diagnose the recent activation of *Toxoplasma*, but no studies have been conducted, about this issue, on leukemia yet (19, 20, 31).

In the present study, one positive case was detected by using the IgG avidity ELISA test that indicated a new infection with *Toxoplasma*. Therefore, avidity testing is very useful for differential diagnosis and timely treatment of active toxoplasmosis.

Toxoplasmosis is not considered as a main risk factor in patients with acute leukemia and lymphoma, considering the possibility of a new infection in patients with immunodeficiency and negative serum and also secondary toxoplasmosis in patients with positive serum and the subsequent brain complications, it is necessary to perform specialized pre-treatment tests in these patients as a group of immunocompromised patients who undergo chemotherapy and these tests should be considered in therapeutic protocols. The limitations of this study included the patient’s post-treatment follow-up status as some patients died, and some did not return to the study due to their long physical distances.

## Conclusion

*T. gondii* infection is very common among patients with acute leukemia in Tabriz, Iran. The results of the present study showed that *Toxoplasma* has the potential to develop new infection in *Toxoplasma* seronegative patients with leukemia and lymphoma. Therefore, due to the risk of secondary toxoplasmosis in patients with positive serum and subsequent occurrence of brain complications in individuals with negative serum, screening of patients with leukemia and lymphoma, as a group of immunosuppressed patients who undergo chemotherapy, is necessary. Considering the high prevalence of positive cases in this study, oncologists should consider specialized tests for toxoplasmosis before, during, and after chemotherapy.
